# Alteration in Nasopharyngeal Microbiota Profile in Aged Patients with COVID-19

**DOI:** 10.3390/diagnostics11091622

**Published:** 2021-09-05

**Authors:** Ravindra Kolhe, Nikhil Shri Sahajpal, Sagar Vyavahare, Akhilesh S. Dhanani, Satish Adusumilli, Sudha Ananth, Ashis K. Mondal, G. Taylor Patterson, Sandeep Kumar, Amyn M. Rojiani, Carlos M. Isales, Sadanand Fulzele

**Affiliations:** 1 Department of Pathology, Augusta University, Augusta, GA 30912, USA; rkolhe@augusta.edu (R.K.); NSAHAJPAL@augusta.edu (N.S.S.); SANANTH@augusta.edu (S.A.); AMONDAL@augusta.edu (A.K.M.); AROJIANI@augusta.edu (A.M.R.); 2 Department of Cellular Biology and Anatomy, Augusta University, Augusta, GA 30912, USA; SVYAVAHARE@augusta.edu (S.V.); SKUMAR1@augusta.edu (S.K.); 3 Department of Pharmacology, Dalhousie University, Halifax, NS B3H 4R2, Canada; Akhilesh.Dhanani@Dal.Ca; 4 Department of Pathology, University of Notre Dame, Notre Dame, IN 46556, USA; sadusumi@nd.edu; 5 Department of Orthopedics, Augusta University, Augusta, GA 30912, USA; GPATTERSON1@augusta.edu (G.T.P.); CISALES@augusta.edu (C.M.I.); 6 Department of Pathology, Penn State University, State College, PA 16802, USA; 7 Department of Medicine, Augusta University, Augusta, GA 30912, USA; 8 Center for Healthy Aging, Augusta University, Augusta, GA 30912, USA

**Keywords:** microbiota, nasopharyngeal profile in aged patients with COVID-19 infection and severity

## Abstract

Severe acute respiratory syndrome coronavirus 2 (SARS-Cov-2) is an infectious virus that causes coronavirus disease 2019 (COVID-19) transmitted mainly through droplets and aerosol affecting the respiratory tract and lungs. Little is known regarding why some individuals are more susceptible than others and develop severe symptoms. In this study, we analyzed the nasopharyngeal microbiota profile of aged patients with COVID-19 (asymptomatic vs. symptomatic) vs. healthy individuals. We examined the nasopharynx swab of 84 aged-matched patients, out of which 27 were negative asymptomatic (NegA), 30 were positive asymptomatic (PA), and 27 patients were positive symptomatic (PSY). Our analysis revealed the presence of abundant Cyanobacterial taxa at phylum level in PA (*p*-value = 0.0016) and PSY (*p*-value = 0.00038) patients along with an upward trend in the population of Litoricola, Amylibacter, Balneola, and Aeromonas at the genus level. Furthermore, to know the relationship between the nasal microbiota composition and severity of COVID-19, we compared PA and PSY groups. Our data show that the nasal microbiota of PSY patients was significantly enriched with the signatures of two bacterial taxa: Cutibacterium (*p*-value = 0.045) and Lentimonas (*p*-value = 0.007*)*. Furthermore, we also found a significantly lower abundance of five bacterial taxa, namely: Prevotellaceae (*p*-value = 7 × 10^−6^), Luminiphilus (*p*-value = 0.027), Flectobacillus (*p*-value = 0.027), Comamonas (*p*-value = 0.048), and Jannaschia (*p*-value = 0.012) in PSY patients. The dysbiosis of the nasal microbiota in COVID-19 positive patients might have a role in contributing to the severity of COVID-19. The findings of our study show that there is a strong correlation between the composition of the nasal microbiota and COVID-19 severity. Further studies are needed to validate our finding in large-scale samples and to correlate immune response (cytokine Strome) and nasal microbiota to identify underlying mechanisms and develop therapeutic strategies against COVID-19.

## 1. Introduction

In little more than three months after the first discovery of the SARS-Cov-2 virus in Hubei, Wuhan, China, in the early days of 2020, the virus quickly spread across the globe. The novel nature of COVID-19 has left our immune systems completely vulnerable to disease. Now, at the time of writing, COVID-19 has been documented to have infected over 146 million individuals, while also claiming the lives of well over three million people, according to Johns Hopkins Coronavirus Resource Center [1]. Although the case fatality rate is orders of magnitude lower than other infectious diseases, such as Ebola and SARS [2,3], the high transmissibility and respiratory nature of the virus are great concerns.

Due to COVID-19 being a new disease, research and information about the symptoms, side effects, prognosis, and treatment are very limited, and gathering more is an ongoing process. However, since the virus began spreading worldwide last year, our knowledge regarding many aspects of the disease has vastly improved. For instance, we now know that the risk of hospitalization and mortality is greatly increased in individuals aged 65+ and those who have underlying conditions, including hypertension, diabetes, and other metabolic disorders [4,5,6]. The most striking feature of COVID-19 positive patients is the wide clinical spectrum, with the majority remaining asymptomatic or exhibiting mild cold-like symptoms, while others have severe viral pneumonia requiring prolonged hospitalization in intensive care unit stay or resulting in death. Reasons as to why some individuals exhibit mild to severe symptoms, while others are solely asymptomatic, are still unknown. Recently, several studies have suggested that bacterial composition of the nasal canal can have a drastic influence upon the development of respiratory infections and the severity of symptoms that accompany the illness [7,8,9,10,11]. It has also been reported that an increase in species richness in an individual’s nasal microbiota is correlated to fewer and less severe symptoms during rhinovirus infections [8,9].

In the last several decades, clinical studies have uncovered the critical roles played by the microflora population in directing and regulating various biological systems throughout the body. Diseases such as chronic inflammatory bowel syndrome, peptic ulcers, and viral diseases have all been recently linked to significant changes in the microbiota of the gut, nasal and oral cavity. Considering COVID-19′s similar characteristics to many viruses, it seems plausible to think that a patient’s nasal microbiota may play a key role in determining the degree to which they exhibit symptoms. Therefore, this study aimed to compare the nasal bacteriome in patients with severe COVID-19 with that of asymptomatic COVID-19 and healthy individuals to document the changes in the diversity and composition of bacteriome between the three cohorts.

## 2. Methods

Study site, ethics, and collection of patient specimens: The samples were processed under an approved HAC by the IRB Committee A (IRB REGISTRATION # 611298), Augusta University, GA. All methods were performed in accordance with the relevant guidelines and regulations. No consent was needed because it was a retrospective study. Based on the IRB approval, all personal health information (PHI) was removed and all data were anonymized before accessing for the study. The study evaluated 84 (49–78-year-old) patients’ nasopharyngeal (NPS) clinical specimens, which included 27 negative asymptomatic (NegA), 30 positive COVID-19 asymptomatic (PA), and 27 positive COVID-19 symptomatic (PSY) samples matched by age, gender, and race (Supplementary Table S1). NPS samples collected in either healthcare or community setting were tested using the FDA-EUA approved SARS-CoV-2 assay (PerkinElmer, Inc., Waltham, MA, USA). As a standard protocol, NPS samples were collected by a healthcare worker using a sterile flocked swab placed in a sterile tube containing the viral transport medium (VTM) (Becton Dickinson, USA, cat. no. 22053). All samples were transported to the SARS-CoV-2 testing facility at Augusta University, GA, within 24 h of sample collection, for further processing.

FDA-EUA approved assay for the detection of SARS-CoV-2 (SARS-specific assay): The assay is based on nucleic acid extraction followed by TaqMan-based RT-PCR assay to conduct in vitro transcription of SARS-CoV-2 RNA, DNA amplification, and fluorescence detection (FDA-EUA assay by PerkinElmer, Inc., Waltham, MA, USA, cat. no. 2019-nCoV-PCR-AUS) [12]. The assay targets specific genomic regions of SARS-CoV-2: nucleocapsid (*N*) and *ORF1ab* gene. The TaqMan probes for the two amplicons are labelled with FAM and ROX fluorescent dyes, respectively, to generate target-specific signals. The assay includes an RNA internal control (IC, bacteriophage MS2) to monitor processes from nucleic acid extraction to fluorescence detection. The IC probe is labelled with VIC fluorescent dye to differentiate its fluorescent signal from SARS-CoV-2 targets. The samples were resulted as positive or negative based on the Ct values specified by the manufacturer. For a detailed method, please refer to Sahajpal, NS, et al. [12].

Microbial Community Profiling Using 16S rRNA Amplicon Sequencing: The DNA isolation kit (Qiagen, USA) was used to isolate genomic DNA samples from NPS samples as per manufacturer’s protocol. The isolated genomic DNA was quantified with nanodrop and stored at -20 C. The DNA samples were shipped to Integrated Microbiome Resource (Dalhousie University, Halifax, NS, Canada) for 16S V4-V5 region amplicon sequencing on Illumina MiSeq, resulting in 300 bp paired-end reads.

Bioinformatics Analysis: We processed the data using the Microbiome Helper workflow based on QIIME2 (version 2020.8) [13,14]. Briefly, the raw data were trimmed to remove primer sequence using cutadapt, and VSEARCH was used to join forward and reverse reads before quality filtering at default settings. Deblur was employed to identify amplicon sequence variants (ASVs) with default parameters and p-trim-length set to 357 bp. Taxonomy to ASVs was assigned using a Naive Bayes approach implemented in the scikit-learn Python library and the SILVA database. Rare, contaminant, and unclassified ASVs were removed from downstream analysis. All statistical analysis and visualizations were conducted in R (version 3.6.3) using phyloseq, vegan, and ggplot2 packages. ASV counts were transformed to relative abundance for comparison of taxonomic abundance between different sample groups using multiple group Kruskal–Wallis test and two-group comparison with Wilcoxon test. Statistical differences in beta diversity were examined with PERMANOVA and PERMDISP tests using QIIME 2 plugins and Vegan package in R. Differential abundance of taxa between groups was identified using the linear discriminant analysis effect size (LEfSe).

## 3. Results and Discussion

It is well documented that certain microorganisms in the nasal microbiota can influence the immune response in patients suffering from respiratory infections [15]. The relationship between nasal microbiota and severity of SARS-CoV-2 infection is not yet known. Our study’s aim was to identify the correlation between nasal microbiota and severity of SARS-CoV-2 in aged patients. First, we confirmed COVID-19 positive and negative samples using the standard RT-PCR method recommended by the CDC [12]. For our study, we used aged-matched 84 (48–70-year-old) patients nasopharyngeal swab samples, out of which 27 were COVID-19 negative (NegA) and collected randomly from community without any symptoms, 30 were COVID-19 positive but asymptomatic (PA), and 27 samples were COVID-19 positive symptomatic (PSY) (mild to severe symptoms) of differing gender and race (Table S1). We found surprising results: out of 27 samples from the NegA group, 2 samples were low reads, and out of 30 samples from the PA group, 4 samples were low reads, whereas 14 samples from the COVID-19 positive symptomatic (PSY) group were low reads (Table 1). This drastic difference suggests a low level of the bacterial population in COVID-19 positive symptomatic (PSY) nasopharyngeal samples compared to the other two groups. This might be due to a washed-off nasal microbiota because of running nose and sneezing, as these are prominent symptoms of patients with COVID-19 [16]. The other logical explanation is that patients with low microbiota content might be at higher risk of developing severe symptoms.

**Figure 1:**
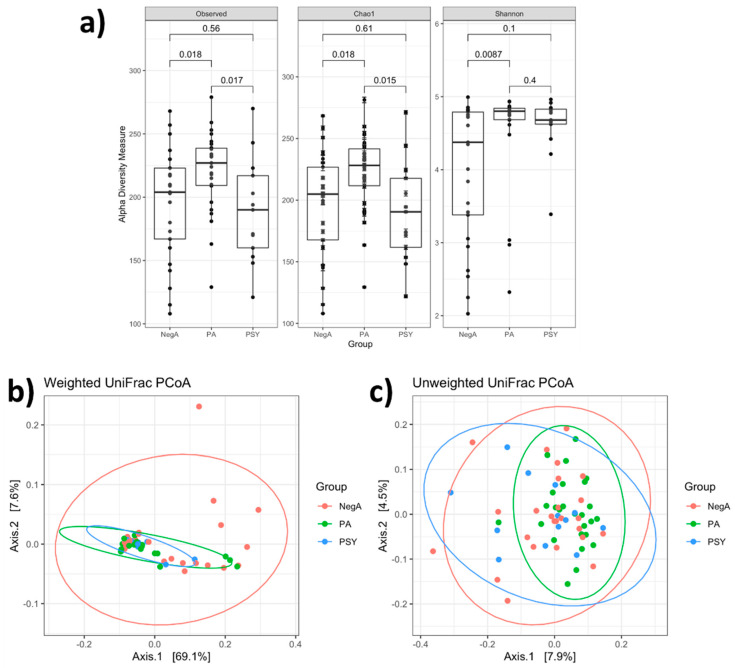
Alpha and beta diversity plots to visualize the difference in nasal microbiota in COVID-19 positive asymptomatic (PA) and COVID-19 positive symptomatic (PSA) patients compared to negative patients (NegA). (**a**) Alpha diversity measures with the most common indices. p-values were obtained using Wilcoxon test. The line in the middle of the box, bound of the box, and whiskers represent the median, 25th–75th percentiles, and min-to-max values, respectively. First two axes of PCoA show (**b**) unweighted and (**c**) weighted UniFrac distances from the beta diversity of study groups. Each dot represents an individual sample. Beta diversity depicting differences in the bacterial community among groups was tested by pairwise permutational multivariate analysis of variation (PERMANOVA; Adonis function in vegan).

Inter-individual beta diversity weighted UniFrac dissimilarity revealed that the microbial community of the PA group (PERMANOVA, R2  = 0.081, *p* < 0.02) and the PSY group (PERMANOVA, R2  = 0.099, *p* < 0.026) clustered apart from that of the NegA group (Figure 1b). We also found a significant dissimilarity in unweighted UniFrac distances between the PA group and the NegA group (PERMANOVA, R2  =  0.026, *p* < 0.035) (Figure 1c). In the case of weighted UniFrac dissimilarity, the significant differences were partly attributed to the different dispersions between the NegA group along with the PA group (PERMDISP, *p*  = 0.016) and the PSY group (PERMDISP, *p* = 0.011). However, the difference in dispersion between the NegA group and the PA group was not significant (PERMDISP, *p* = 0.231) in the case of unweighted UniFrac dissimilarity (Figure 1).

**Figure 2:**
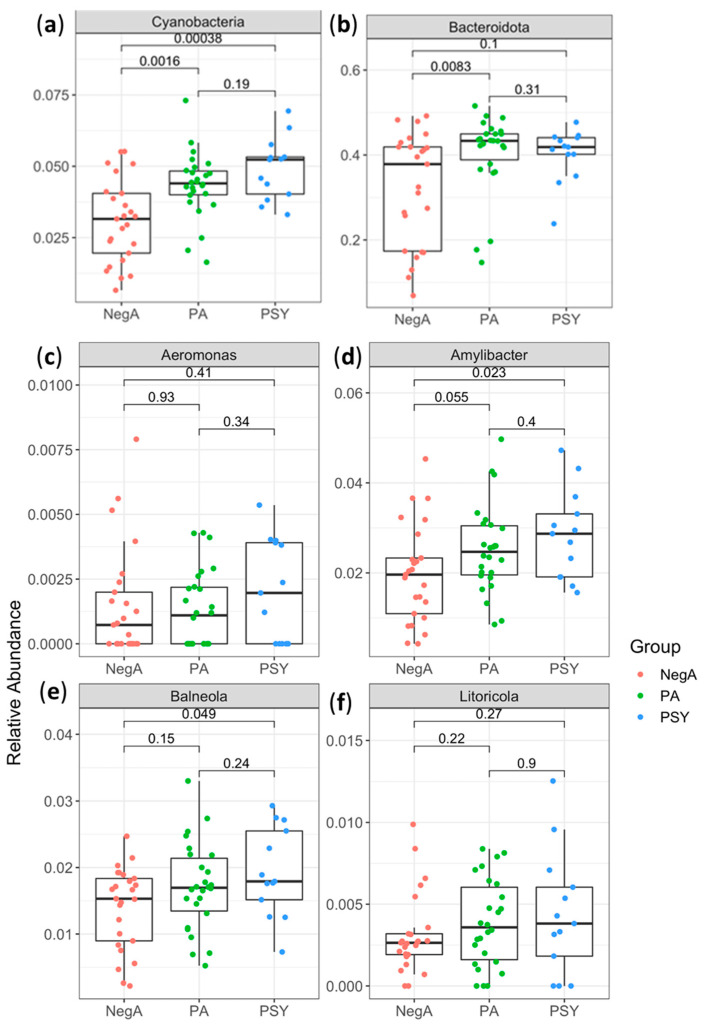
Change in composition of the nasal microbiota at the phylum in COVID-19 positive asymptomatic (PA) and COVID-19 positive symptomatic (PSA) patients compared to negative patients (NegA). The (**a**) Cyanobacteria show significantly high abundance in PA and PSA compared to NegA, and (**b**) Bacteroidota, (**c**) Aeromonas, (**d**) Amylibacter, (**e**) Balneola, and (**f**) Litaricola showed a trend of higher abundance. Within each box, horizontal black lines denote median values; boxes extend from the 25th to the 75th percentile of each group’s distribution of values; vertical extending lines denote adjacent values (i.e., the most extreme values within 1.5 interquartile range of the 25th and 75th percentile of each group); dots denote observations outside the range of adjacent values; and *p*-values were calculated using multiple-group Kruskal–Wallis test and two-group Wilcoxon test.

To identify nasal bacteria associated with SARS-CoV-2 infection, we compared COVID-19 negative samples with COVID-19 positive samples with or without symptoms. In our study, we found a significant increase in the bacterial taxa of Cyanobacteria at phylum level in COVID-19 positive asymptomatic (*p*-value = 0.0016*)* and symptomatic (*p*-value = 0.00038) patients (Figure 2). Furthermore, we found an upward trend in the population of Bacteroidota, Litoricola, Amylibacter, Balneola, and Aeromonas at the genus level (Figure 2) in the COVID-19 infected patients, although we did not find any significant differences in Shannon Index, Alpha Diversity, Observed Species, or Bacterial Richness between the COVID-19 negative and positive patient groups.

Normal human microbial flora in humans is composed of mainly six phyla: Actinobacteria, Bacteroidetes, Cyanobacteria, Firmicutes, Fusobacteria, and Proteobacteria [17]. Significant increase in the bacterial taxa of Cyanobacteria at phylum level in COVID-19 positive patients implies that Cyanobacteria play a pivotal role in regulating the immune response. Previously, a study carried out by Lee et al. in asthmatic patients revealed that Cyanobacteria, Bacteroides, and Fusobacteria were significantly more abundant in asthmatic patients than in the healthy controls [18] Recently, De Maio et al. demonstrated that the nasal microbiota composition of the patients affected with SARS-Cov-2 mainly consisted of Bacteroides, Proteobacteria, Actinobacteria, Firmicutes, and Fusobacteria [19]. The findings of our study align with their studies, as we also found an increasing trend in the population of these bacteria. Microbiota is an integral part of the defense mechanism of the host, helping to prevent or reduce viral infection by regulating immune response [20], but viral infection can also alter the microbiota composition of the host and further influence the infectivity [21]. The findings of our study imply that the nasal microbiota composition of patients might have undergone some alterations in response to COVID-19 infection, or patients with above mentioned bacterial taxa are more susceptible to COVID-19 infections.

**Figure 3:**
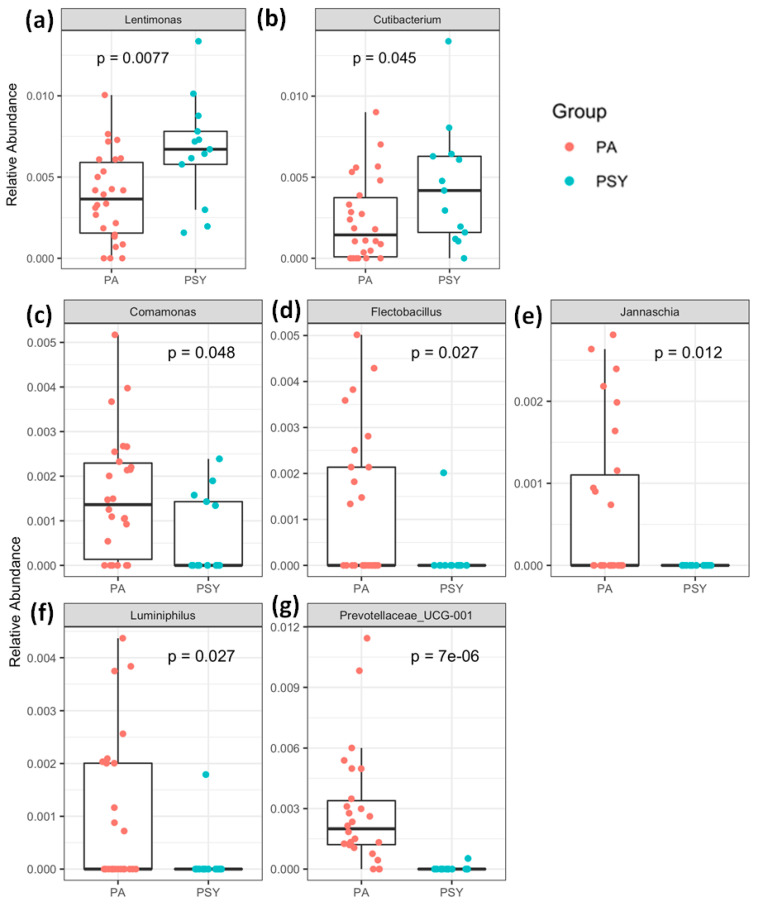
Dysbiosis of the nasal microbiota in COVID-19 symptomatic patients. (**a**) Lentimonas (*p* value = 0.007) and (**b**) Cutibacterium (*p* value = 0.045) bacteria elevated at the genus level, whereas (**c**) Comamonas (*p* value = 0.048), (**d**) Flectobacillus (*p* value = 0.027), (**e**) Jannaschia (*p* value = 0.012), (**f**) Luminiphilus (*p* value = 0.027), and (**g**) Prevotellaceae (*p* value = 7 × 10^−6^) show significantly low abundance in PSA compared to PA. Within each box, horizontal black lines denote median values; boxes extend from the 25th to the 75th percentile of each group’s distribution of values; vertical extending lines denote adjacent values (i.e., the most extreme values within 1.5 interquartile range of the 25th and 75th percentile of each group); dots denote observations outside the range of adjacent values; and *p*-values were calculated using multiple-group Kruskal–Wallis test and two-group Wilcoxon test.

**Figure 4:**
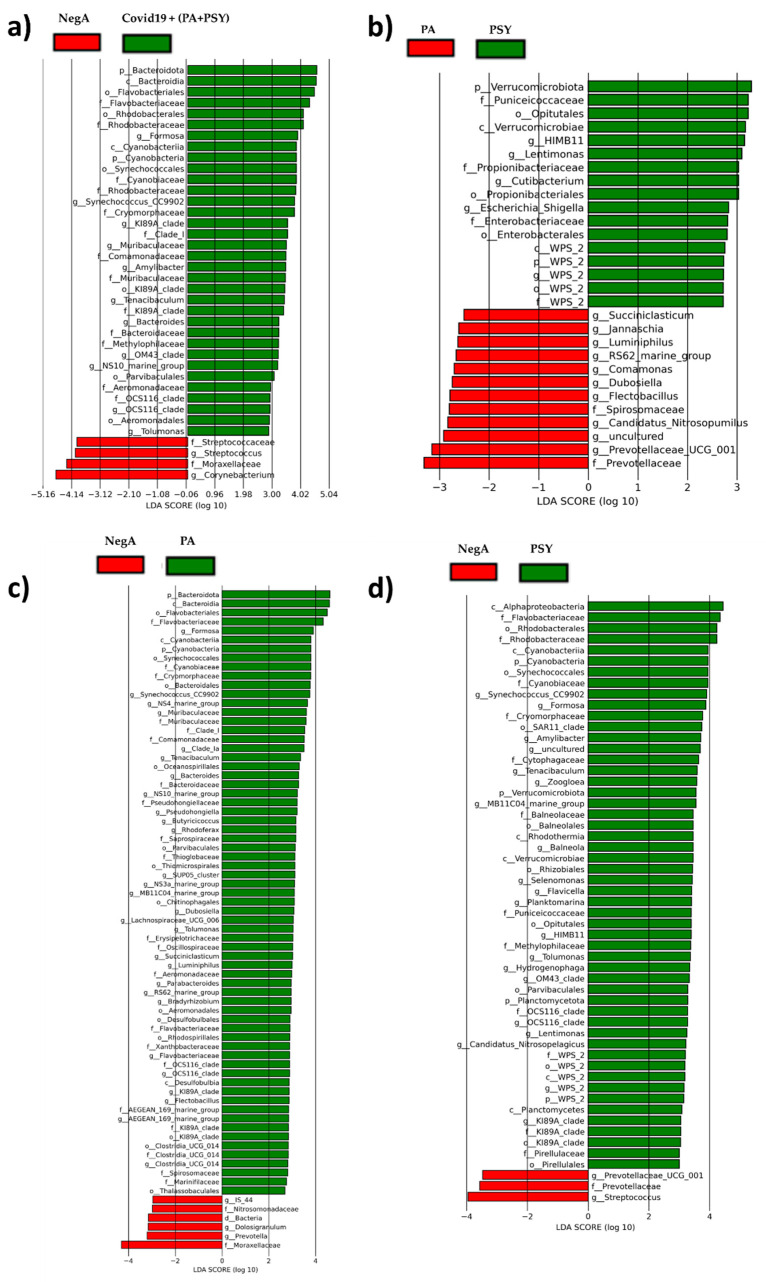
Histogram of the linear discriminant analysis (LDA) scores for differentially abundant taxonomic features between (**a**) NegA (COVID-19 –ve) (red) and COVID-19 +ve (PSA and PA) (green), (**b**) PA (red) and PSA (green), (**c**) NegA (COVID-19 –ve) (red) and PA (green), and (**d**) NegA (COVID-19 –ve) (red) and PSA (green). The threshold on the logarithmic LDA score for discriminative features was set to 2.0 for LEfSe analysis.

To investigate the relationship between the severity of COVID-19 and the composition of the nasal microbiota, we compared COVID-19 positive asymptomatic (PA) and COVID-19 positive symptomatic (PSY) groups. Our analyses revealed that PSY patients were enriched with the signatures of two bacteria:, Cutibacterium (*p* value = 0.045) and Lentimonas (*p* value < 0.007) (Figure 3). Cutibacterium is a commensal micro-organism that inhabits mucosal surfaces. Generally, Cutibacterium is considered as taxa that promote sinus health and is thought to be the front-line defense system that acts against a variety of bacterial, viral, and fungal infections [21]. Although few studies have reported it, Cutibacterium is enriched in certain respiratory disease conditions [22]. Till data, not much information is available about Lentimonas in viral diseases and res-piratory tract infections. In our study, we also found five signature bacteria were significantly reduced in PSY patients, namely: Prevotellaceae (*p*-value = 7 × 10^−6^), Luminiphilus (*p* value = 0.027), Flectobacillus (*p* value = 0.027), Comamonas (*p* value = 0.048), and Jannaschia (*p* value = 0.012) (Figure 3). Prevotellaceae found in the microbiota of the head and neck region [23] were significantly decreased in influenza virus-infected patients [24]. The Proteobacteria is a commensal organism that resides in anterior nares in the upper respiratory tract in healthy adults [15]. This bacteria was at a significantly low level in PSY patients, suggesting a role in increased susceptibility to COVID-19 infection. Similar bacterial taxa community compositions were also observed after linear discriminant effect size analysis (LEfSe) (Figure 4) between the groups. The findings of our study suggest that alterations in the microbiota in COVID-19 positive symptomatic patients might have a role in regulating immune response to fight against severe infections. Dysbiosis of the nasal microbiota might be one of the reasons for the increased susceptibility and severity of COVID-19 infection.

Overall, our study showed a strong association between the composition of nasal microbiota and SARS-CoV-2 infection and severity. The microbiota composition varies from person to person and may account for the inter-individual variability in response to COVID-19. This might be one of the reasons why some patients with the SARS-CoV-2 virus can develop mild to severe symptoms and some patients tend to remain asymptomatic. Our study found a correlation between change in nasal microbiota and SARS-CoV-2 infection. The main limitations of our study are the relatively small sample size and our patient selection including only the aged population. Moreover, we did not compare the results based on gender and race due to the limited sample size; however, the samples were gender- and race-matched to minimize bias. Overall, our preliminary results show that the nasal microbiota profile of the patients affected with COVID-19 provides a piece of insightful information that can help in developing both biomarkers to assess the severity of disease and new therapeutic strategies to mitigate negative outcomes for patients. Further studies need to be carried out as to how the nasal microbiota can be manipulated to identify the mechanisms by which it can enhance immune health, prevent or treat SARS-CoV-2 infections, and build immunity.

## Figures and Tables

**Table 1:** Groupwise amplification of reads.

Sample Type	Sample Processed	Amplicons/Reads
NegA	27	25
PA	30	26
PSY	27	13

## Data Availability

All relevant data is made available in the manuscript and supplementary files.

## Supplementary Materials

The following are available online at https://www.mdpi.com/article/10.3390/diagnostics11091622/s1, Table S1: Detailed information of the samples used for the current study.
