# Supplementary material for: Alteration in Nasopharyngeal Microbiota Profile in Aged Patients with COVID-19
Source: Diagnostics (Basel). 2021 Sep 5;11(9):1622. doi: 10.3390/diagnostics11091622 (PMC8467337; doi:10.3390/diagnostics11091622)
Supplement: Supplementary file 1 [file diagnostics-11-01622-s001.zip › diagnostics-1321994-supplementary.pdf]

Supplementary Table S1. Detailed information of the samples used for the current study.

Sample ID	Age	Sex	Ethnicity
	**Negative Asymptomatic (NegA)**		
NA-1.	61 years	Female	African American
NA-2.	58 years	Female	African American
NA-3.	59 years	Female	African American
NA-4.	65 years	Female	African American
NA-5.	67 years	Female	African American
NA-9.	59 years	Female	Caucasian
NA-15.	70 years	Male	African American
NA-16.	69 years	Male	African American
NA-17.	70 years	Male	African American
NA-18.	65 years	Male	Caucasian
NA-19.	57 years	Male	Caucasian
NA-20.	59 years.	Male	Caucasian
NA-22.	64 year	Male	Caucasian
NA-24.	58 years	Female	African American
NA-25.	73 years	Female	Caucasian
NA-26.	64 years	Female	Caucasian
NA-27.	60 years	Female	Caucasian
NA-29.	51 years	Female	Caucasian
NA-30.	55 years	Female	Caucasian
NA-31.	58 years	Male	African American
NA-32.	52 Year	Male	African American
NA-34.	62 Year	Male	African American
NA-35.	64 Year	Male	African American
NA-33.	57 Year	Male	Caucasian
NA-36.	63 years	Male	Caucasian
NA-37.	68 years	Male	Caucasian
NA-38.	52 years	Male	Caucasian
	**Positive Asymptomatic (PA)**		
PA-2	75 years	Female	African American
PA-3	69 years	Female	African American
PA-4	61 years	Female	African American
PA-5	78 years	Female	African American
PA-6	69 years	Female	African American
PA-8	69 years	Female	Caucasian
PA-9	59 years	Female	Caucasian
PA-10	63 years	Female	Caucasian
PA-11	61 years	Female	Caucasian
PA-12	60 years	Female	Caucasian
PA-13	62 years	Female	Caucasian
PA-14	64 years	Female	Caucasian
PA-15	60 years	Male	African American
PA-16	72 years	Male	African American
PA-17	64 years	Male	African American
PA-18	59 years	Male	African American
PA-19	60 years	Male	African American
PA-20	63 years	Male	African American
PA-21	55 years	Male	African American

PA-22	65 years	Male	Caucasian
PA-23	72 years	Male	Caucasian
PA-25	64 years	Male	Caucasian
PA-26	50 years	Male	Caucasian
PA-27	57 years	Male	Caucasian
PA-28	53 years	Female	African American
PA-29	67 years	Female	Caucasian
PA-30	73 years	Male	African American
PA-31	71 years	Male	Caucasian
PA-32	62 years	Male	Caucasian
PA-33	58 years	Male	Caucasian
**Positive Symptomatic (PSY)**			
PSY-1	66 Year	Female	Caucasian
PSY-2	57 Year	Female	Caucasian
PSY-3	54 Year	Female	Caucasian
PSY-4	51 year	Female	Caucasian
PSY-5	76 year	Female	Caucasian
PSY-6	59 year	Female	Caucasian
PSY-7	48 year	Female	African American
PSY-8	63 year	Female	African American
PSY-9	55 year	Female	African American
PSY-10	62 year	Female	African American
PSY-11	57 year	Female	African American
PSY-12	51 year	Male	Caucasian
PSY-13	55 year	Male	Caucasian
PSY-14	48 year	Male	Caucasian
PSY-16	74 year	Male	African American
PSY-17	50 year	Male	African American
PSY-20	50 year	Male	African American
PSY-21	49 Year	Male	African American
PSY-22	47 Year	Male	African American
PSY-23	61 Year	Female	Caucasian
PSY-24	72 Year	Female	African American
PSY-25	51 year	Female	African American
PSY-26	56 Year	Male	Caucasian
PSY-27	69 Year	Male	Caucasian
PSY-28	54 Year	Male	Caucasian
PSY-29	51 Year	Male	Caucasian
PSY-30	72 year	Male	African American
